# Supplementary material for: Modeling glioblastoma heterogeneity as a dynamic network of cell states
Source: Mol Syst Biol. 2021 Sep 16;17(9):e10105. doi: 10.15252/msb.202010105 (PMC8444284; doi:10.15252/msb.202010105)
Supplement: Supplementary file 6 — Source Data for Figure 5 [file MSB-17-e10105-s004.zip › Figure5A_sourcedata/GSEA_3017/hallmarks_stateF.GseaPreranked.1621934628066/HALLMARK_E2F_TARGETS.html]

Details for gene set HALLMARK\_E2F\_TARGETS[GSEA]

|  || Dataset | state33017 |
| Phenotype | NoPhenotypeAvailable |
| Upregulated in class | na\_neg |
| GeneSet | HALLMARK\_E2F\_TARGETS |
| Enrichment Score (ES) | -0.74451053 |
| Normalized Enrichment Score (NES) | -4.984513 |
| Nominal p-value | 0.0 |
| FDR q-value | 0.0 |
| FWER p-Value | 0.0 |
Table: GSEA Results Summary

  

Fig 1: Enrichment plot: HALLMARK\_E2F\_TARGETS      
 Profile of the Running ES Score & Positions of GeneSet Members on the Rank Ordered List

  

| PROBE | GENE SYMBOL | GENE\_TITLE | RANK IN GENE LIST | RANK METRIC SCORE | RUNNING ES | CORE ENRICHMENT || 1 | CDKN1A |  |  | 4 | 2.089 | 0.0229 | No |
| 2 | TFRC |  |  | 270 | 0.537 | -0.0953 | No |
| 3 | PPM1D |  |  | 856 | 0.334 | -0.3661 | No |
| 4 | PMS2 |  |  | 1389 | 0.258 | -0.6129 | No |
| 5 | RPA2 |  |  | 1490 | -0.256 | -0.6569 | No |
| 6 | NOLC1 |  |  | 1542 | -0.270 | -0.6776 | No |
| 7 | PSIP1 |  |  | 1564 | -0.275 | -0.6842 | No |
| 8 | CDKN2C |  |  | 1593 | -0.285 | -0.6940 | No |
| 9 | PRKDC |  |  | 1594 | -0.285 | -0.6906 | No |
| 10 | RAD1 |  |  | 1613 | -0.289 | -0.6957 | No |
| 11 | POLA2 |  |  | 1622 | -0.291 | -0.6960 | No |
| 12 | GINS1 |  |  | 1634 | -0.295 | -0.6976 | No |
| 13 | GSPT1 |  |  | 1653 | -0.303 | -0.7025 | No |
| 14 | NUP107 |  |  | 1683 | -0.313 | -0.7124 | No |
| 15 | CTPS1 |  |  | 1692 | -0.316 | -0.7125 | No |
| 16 | MRE11 |  |  | 1693 | -0.316 | -0.7087 | No |
| 17 | MCM3 |  |  | 1738 | -0.335 | -0.7254 | No |
| 18 | PLK4 |  |  | 1740 | -0.336 | -0.7219 | No |
| 19 | PAICS |  |  | 1757 | -0.342 | -0.7254 | No |
| 20 | RAN |  |  | 1772 | -0.350 | -0.7278 | No |
| 21 | LYAR |  |  | 1774 | -0.350 | -0.7241 | No |
| 22 | LIG1 |  |  | 1797 | -0.361 | -0.7302 | No |
| 23 | XRCC6 |  |  | 1825 | -0.377 | -0.7384 | No |
| 24 | BARD1 |  |  | 1839 | -0.384 | -0.7400 | Yes |
| 25 | MMS22L |  |  | 1846 | -0.387 | -0.7382 | Yes |
| 26 | KIF18B |  |  | 1860 | -0.393 | -0.7397 | Yes |
| 27 | E2F8 |  |  | 1864 | -0.396 | -0.7364 | Yes |
| 28 | EXOSC8 |  |  | 1865 | -0.397 | -0.7317 | Yes |
| 29 | POLD2 |  |  | 1868 | -0.398 | -0.7279 | Yes |
| 30 | SYNCRIP |  |  | 1877 | -0.403 | -0.7269 | Yes |
| 31 | SRSF1 |  |  | 1879 | -0.405 | -0.7226 | Yes |
| 32 | ASF1B |  |  | 1897 | -0.419 | -0.7256 | Yes |
| 33 | MXD3 |  |  | 1905 | -0.428 | -0.7238 | Yes |
| 34 | HMGA1 |  |  | 1915 | -0.433 | -0.7229 | Yes |
| 35 | POLD3 |  |  | 1918 | -0.436 | -0.7187 | Yes |
| 36 | DIAPH3 |  |  | 1924 | -0.439 | -0.7158 | Yes |
| 37 | SNRPB |  |  | 1937 | -0.447 | -0.7162 | Yes |
| 38 | STAG1 |  |  | 1943 | -0.450 | -0.7132 | Yes |
| 39 | NAP1L1 |  |  | 1951 | -0.455 | -0.7111 | Yes |
| 40 | NOP56 |  |  | 1953 | -0.456 | -0.7062 | Yes |
| 41 | RFC2 |  |  | 1954 | -0.457 | -0.7008 | Yes |
| 42 | DDX39A |  |  | 1957 | -0.458 | -0.6963 | Yes |
| 43 | DNMT1 |  |  | 1969 | -0.465 | -0.6960 | Yes |
| 44 | CIT |  |  | 1978 | -0.474 | -0.6941 | Yes |
| 45 | POP7 |  |  | 1981 | -0.474 | -0.6894 | Yes |
| 46 | CSE1L |  |  | 1985 | -0.479 | -0.6852 | Yes |
| 47 | NASP |  |  | 1988 | -0.481 | -0.6804 | Yes |
| 48 | TIMELESS |  |  | 1989 | -0.482 | -0.6747 | Yes |
| 49 | MCM4 |  |  | 1993 | -0.487 | -0.6704 | Yes |
| 50 | SPAG5 |  |  | 1997 | -0.490 | -0.6660 | Yes |
| 51 | TRA2B |  |  | 1998 | -0.490 | -0.6602 | Yes |
| 52 | TK1 |  |  | 2003 | -0.497 | -0.6562 | Yes |
| 53 | XPO1 |  |  | 2007 | -0.500 | -0.6516 | Yes |
| 54 | CTCF |  |  | 2010 | -0.502 | -0.6467 | Yes |
| 55 | CHEK1 |  |  | 2013 | -0.504 | -0.6416 | Yes |
| 56 | SMC1A |  |  | 2021 | -0.513 | -0.6388 | Yes |
| 57 | RANBP1 |  |  | 2024 | -0.517 | -0.6337 | Yes |
| 58 | JPT1 |  |  | 2036 | -0.525 | -0.6326 | Yes |
| 59 | HELLS |  |  | 2038 | -0.528 | -0.6268 | Yes |
| 60 | CDCA3 |  |  | 2041 | -0.529 | -0.6215 | Yes |
| 61 | TRIP13 |  |  | 2044 | -0.530 | -0.6162 | Yes |
| 62 | ILF3 |  |  | 2052 | -0.537 | -0.6131 | Yes |
| 63 | KIF22 |  |  | 2053 | -0.541 | -0.6067 | Yes |
| 64 | STMN1 |  |  | 2063 | -0.567 | -0.6042 | Yes |
| 65 | RACGAP1 |  |  | 2072 | -0.579 | -0.6011 | Yes |
| 66 | GINS4 |  |  | 2075 | -0.586 | -0.5951 | Yes |
| 67 | AURKB |  |  | 2079 | -0.598 | -0.5895 | Yes |
| 68 | USP1 |  |  | 2084 | -0.612 | -0.5841 | Yes |
| 69 | PLK1 |  |  | 2087 | -0.624 | -0.5776 | Yes |
| 70 | NCAPD2 |  |  | 2089 | -0.627 | -0.5707 | Yes |
| 71 | SSRP1 |  |  | 2091 | -0.630 | -0.5637 | Yes |
| 72 | RFC3 |  |  | 2098 | -0.642 | -0.5589 | Yes |
| 73 | BRCA2 |  |  | 2100 | -0.650 | -0.5517 | Yes |
| 74 | PSMC3IP |  |  | 2106 | -0.659 | -0.5462 | Yes |
| 75 | AURKA |  |  | 2110 | -0.673 | -0.5397 | Yes |
| 76 | RPA3 |  |  | 2112 | -0.678 | -0.5321 | Yes |
| 77 | CKS2 |  |  | 2115 | -0.694 | -0.5249 | Yes |
| 78 | CDC20 |  |  | 2117 | -0.695 | -0.5171 | Yes |
| 79 | RAD21 |  |  | 2118 | -0.697 | -0.5088 | Yes |
| 80 | SRSF2 |  |  | 2122 | -0.706 | -0.5019 | Yes |
| 81 | PA2G4 |  |  | 2123 | -0.707 | -0.4935 | Yes |
| 82 | CENPM |  |  | 2125 | -0.709 | -0.4856 | Yes |
| 83 | HNRNPD |  |  | 2126 | -0.713 | -0.4772 | Yes |
| 84 | RAD51AP1 |  |  | 2127 | -0.715 | -0.4687 | Yes |
| 85 | CDCA8 |  |  | 2129 | -0.722 | -0.4606 | Yes |
| 86 | KIF2C |  |  | 2136 | -0.756 | -0.4545 | Yes |
| 87 | BRCA1 |  |  | 2138 | -0.758 | -0.4460 | Yes |
| 88 | MELK |  |  | 2148 | -0.786 | -0.4409 | Yes |
| 89 | SMC3 |  |  | 2150 | -0.800 | -0.4319 | Yes |
| 90 | DEPDC1 |  |  | 2152 | -0.809 | -0.4228 | Yes |
| 91 | DEK |  |  | 2154 | -0.817 | -0.4136 | Yes |
| 92 | DUT |  |  | 2155 | -0.824 | -0.4038 | Yes |
| 93 | TUBB |  |  | 2157 | -0.831 | -0.3945 | Yes |
| 94 | MCM7 |  |  | 2160 | -0.839 | -0.3855 | Yes |
| 95 | HMGB3 |  |  | 2164 | -0.852 | -0.3768 | Yes |
| 96 | SPC24 |  |  | 2166 | -0.853 | -0.3672 | Yes |
| 97 | LBR |  |  | 2173 | -0.870 | -0.3597 | Yes |
| 98 | BUB1B |  |  | 2174 | -0.871 | -0.3494 | Yes |
| 99 | KPNA2 |  |  | 2178 | -0.894 | -0.3402 | Yes |
| 100 | HMMR |  |  | 2179 | -0.895 | -0.3296 | Yes |
| 101 | KIF4A |  |  | 2186 | -0.921 | -0.3215 | Yes |
| 102 | ORC6 |  |  | 2188 | -0.937 | -0.3109 | Yes |
| 103 | LMNB1 |  |  | 2194 | -0.950 | -0.3020 | Yes |
| 104 | UBE2T |  |  | 2196 | -0.965 | -0.2911 | Yes |
| 105 | CDKN3 |  |  | 2199 | -0.979 | -0.2804 | Yes |
| 106 | CCNB2 |  |  | 2200 | -0.983 | -0.2688 | Yes |
| 107 | CKS1B |  |  | 2201 | -0.987 | -0.2571 | Yes |
| 108 | EZH2 |  |  | 2207 | -1.017 | -0.2474 | Yes |
| 109 | SPC25 |  |  | 2208 | -1.019 | -0.2353 | Yes |
| 110 | CENPE |  |  | 2211 | -1.036 | -0.2240 | Yes |
| 111 | MYBL2 |  |  | 2215 | -1.058 | -0.2129 | Yes |
| 112 | CDK1 |  |  | 2225 | -1.153 | -0.2035 | Yes |
| 113 | PTTG1 |  |  | 2226 | -1.198 | -0.1893 | Yes |
| 114 | ANP32E |  |  | 2227 | -1.200 | -0.1751 | Yes |
| 115 | MAD2L1 |  |  | 2228 | -1.204 | -0.1608 | Yes |
| 116 | UBE2S |  |  | 2229 | -1.205 | -0.1466 | Yes |
| 117 | RRM2 |  |  | 2234 | -1.268 | -0.1334 | Yes |
| 118 | DLGAP5 |  |  | 2235 | -1.273 | -0.1184 | Yes |
| 119 | SMC4 |  |  | 2236 | -1.281 | -0.1032 | Yes |
| 120 | ATAD2 |  |  | 2238 | -1.381 | -0.0873 | Yes |
| 121 | TMPO |  |  | 2239 | -1.382 | -0.0710 | Yes |
| 122 | HMGB2 |  |  | 2241 | -1.469 | -0.0540 | Yes |
| 123 | BIRC5 |  |  | 2242 | -1.469 | -0.0367 | Yes |
| 124 | TOP2A |  |  | 2246 | -1.562 | -0.0196 | Yes |
| 125 | MKI67 |  |  | 2252 | -1.892 | 0.0005 | Yes |
Table: GSEA details [plain text format]

  

Fig 2: HALLMARK\_E2F\_TARGETS: Random ES distribution      
 Gene set null distribution of ES for **HALLMARK\_E2F\_TARGETS**
